# Does Tinnitus Depend on Time-of-Day? An Ecological Momentary Assessment Study with the “TrackYourTinnitus” Application

**DOI:** 10.3389/fnagi.2017.00253

**Published:** 2017-08-02

**Authors:** Thomas Probst, Rüdiger C. Pryss, Berthold Langguth, Josef P. Rauschecker, Johannes Schobel, Manfred Reichert, Myra Spiliopoulou, Winfried Schlee, Johannes Zimmermann

**Affiliations:** ^1^Georg-Elias-Müller-Institute for Psychology, Georg-August-University Göttingen Göttingen, Germany; ^2^Department for Psychotherapy and Biopsychosocial Health, Danube University Krems Krems an der Donau, Austria; ^3^Department of Psychiatry and Psychotherapy of the University of Regensburg at Bezirksklinikum Regensburg Regensburg, Germany; ^4^Program in Cognitive and Computational Systems, Georgetown University Washington Washington, DC, United States; ^5^Institute for Advanced Study, Technical University Munich Munich, Germany; ^6^Department of Technical and Business Information Systems, Otto-von-Guericke-University Magdeburg Magdeburg, Germany; ^7^Psychologische Hochschule Berlin Berlin, Germany

**Keywords:** tinnitus, stress, circadian fluctuation, time-of-day, ecological momentary assessment

## Abstract

Only few previous studies used ecological momentary assessments to explore the time-of-day-dependence of tinnitus. The present study used data from the mobile application “TrackYourTinnitus” to explore whether tinnitus loudness and tinnitus distress fluctuate within a 24-h interval. Multilevel models were performed to account for the nested structure of assessments (level 1: 17,209 daily life assessments) nested within days (level 2: 3,570 days with at least three completed assessments), and days nested within participants (level 3: 350 participants). Results revealed a time-of-day-dependence of tinnitus. In particular, tinnitus was perceived as louder and more distressing during the night and early morning hours (from 12 a.m. to 8 a.m.) than during the upcoming day. Since previous studies suggested that stress (and stress-associated hormones) show a circadian rhythm and this might influence the time-of-day-dependence of tinnitus, we evaluated whether the described results change when statistically controlling for subjectively reported stress-levels. Correcting for subjective stress-levels, however, did not change the result that tinnitus (loudness and distress) was most severe at night and early morning. These results show that time-of-day contributes to the level of both tinnitus loudness and tinnitus distress. Possible implications of our results for the clinical management of tinnitus are that tailoring the timing of therapeutic interventions to the circadian rhythm of individual patients (chronotherapy) might be promising.

## Introduction

Tinnitus, the phantom perception of sound (Baguley et al., 2013; Langguth et al., 2013), is perceived by 5.1% up to 42.7% of the population according to a recent review including 39 studies from 16 countries (McCormack et al., 2016). These percentages depend on age (with older persons showing higher prevalence), gender (with male persons showing higher prevalence), and the definition of tinnitus used in the epidemiological study (McCormack et al., 2016). Prevalence rates of tinnitus also have increased over the years (Nondahl et al., 2012; Martinez et al., 2015), and one can speculate about the reasons for this apparent increase. On a neuronal basis, auditory-limbic interactions play a central role in the development of (chronic) tinnitus (Rauschecker et al., 2010; Leaver et al., 2011). In many cases, tinnitus is associated with psychological distress and incapacity for work (Bhatt et al., 2016) resulting in high socio-economic costs (Maes et al., 2013). Cognitive-behavioral therapy (CBT) has proven to have the potential to reduce the burden of tinnitus for the individual (Hesser et al., 2011) as well as the society/economy (Maes et al., 2014). But not all tinnitus patients reach clinically relevant improvements with CBT (e.g., Jasper et al., 2014). Other therapeutic approaches, including pharmacological therapy (Langguth and Elgoyhen, 2012), auditory stimulation (Hobson et al., 2012) or brain stimulation (Langguth and De Ridder, 2013), have only revealed small and inconsistent effects in subgroups of patients. It is assumed that an important reason for the poor treatment response in clinical trials is the heterogeneity of tinnitus (Landgrebe et al., 2012; Baguley et al., 2013), both across patients (inter-individual heterogeneity) as well as within patients over time (intra-individual heterogeneity, see Dauman et al., 2015).

Therefore, it is important to understand the factors that contribute to the heterogeneity of tinnitus. Psychological variables, such as fear-related cognition (e.g., Cima et al., 2011; Kleinstäuber et al., 2013), an accepting stance toward tinnitus (e.g., Weise et al., 2013; Riedl et al., 2015), emotions (e.g., Probst et al., 2016a,b), and avoidance/safety behaviors (e.g., Hesser and Andersson, 2009; Kleinstäuber et al., 2013), have been demonstrated to account for the heterogeneity of tinnitus in several studies (see also the “scientific cognitive-behavioral model of tinnitus”; McKenna et al., 2014). Moreover, recent neuroscience studies imply that the moment-to-moment variability of tinnitus is related to brain oscillatory patterns like the alpha power in temporal regions (Schlee et al., 2014) and time-of-day (Basinou et al., 2017); circadian fluctuations have been shown, for example, in auditory pathway structures related to tinnitus like the cochlea (Meltser et al., 2014) and the inferior colliculus (Park et al., 2016). Another hint for a link between tinnitus and circadian rhythms is provided by findings of reduced tinnitus severity after intake of melatonin (e.g., Pirodda et al., 2010; Ajayi et al., 2014; Miroddi et al., 2015). Furthermore, pain, which shares many similarities with tinnitus (De Ridder et al., 2011; Rauschecker et al., 2015), has been found to underlie circadian variations (e.g., Strian et al., 1989; Gilron and Ghasemlou, 2014; Buttgereit et al., 2015). Furthermore, depression, which overlaps in its pathophysiology with tinnitus (Langguth et al., 2011), is characterized by changes in circadian rhythm (e.g., Germain and Kupfer, 2008; Wirz-Justice, 2008).

The question of whether tinnitus varies systematically over the course of the day, however, has not yet been studied systematically. The limited possibilities of traditional assessment methods to routinely track symptoms in the daily routine have made it rather difficult to validly study whether tinnitus shows time-of-day-dependence. To overcome these limitations of traditional assessment methods, newer technological developments can be used to electronically gather valid daily life data (ecological momentary assessments, EMA; Trull and Ebner-Priemer, 2013, 2014; Adams et al., 2017). Henry et al. (2012), for example, used personal digital assistants (PDA) in a 2-week pilot study with 24 participants to obtain EMA during a 12-h interval (8 a.m.–8 p.m.) and showed that the scores of the 10-item screening version of the Tinnitus Handicap Inventory (THI-S; Newman et al., 2008) were not significantly different between 3-h time blocks (8 a.m.–11 a.m., 11 a.m.–2 p.m., 2 p.m.–5 p.m., and 5 p.m.–8 p.m.). Although Henry et al. (2012) failed to demonstrate time-of-day-dependence of tinnitus, a 2-week pilot smartphone-based study with 20 participants on fluctuations of tinnitus within an 11-h interval (9 a.m.–8 p.m.) suggested that tinnitus does vary within a single day (Wilson et al., 2015). But Wilson et al. (2015) did not report at which time-of-day the participants rated their tinnitus as more or less severe. In another diary study, Flor et al. (2004) reported tinnitus being worst at the beginning of a day, thus supporting the time-of-day-dependence of tinnitus. To our knowledge there are only these three studies with ambivalent results that addressed the time-of-day-dependence of tinnitus in daily life with EMA (Flor et al., 2004; Henry et al., 2012; Wilson et al., 2015).

The current study used EMA from the “TrackYourTinnitus” (TYT) mobile application (Pryss et al., 2015a,b; Schlee et al., 2016) to explore whether tinnitus fluctuates within a 24-h interval (night and upcoming day). Tinnitus is operationalized by two questions in TYT, one question is on tinnitus loudness and the other question on tinnitus distress. Prior research suggested that an assessment of both tinnitus loudness and tinnitus distress is necessary for a comprehensive assessment, since tinnitus loudness and tinnitus distress are only moderately correlated (e.g., Hiller and Goebel, 2007; Wallhäusser-Franke et al., 2012) and processed in different but interconnected brain areas (e.g., Leaver et al., 2012; Ueyama et al., 2013; De Ridder et al., 2014; Vanneste et al., 2014). Therefore, it appears possible that tinnitus loudness and tinnitus distress show either similar or different ups and downs within a 24-h interval. Accordingly, the present study investigated the time-of-day-dependence of tinnitus loudness as well as of tinnitus distress. Moreover, stress is known to be associated with tinnitus (e.g., Hébert et al., 2004; Hébert and Lupien, 2007, 2009; Alsalman et al., 2016) and stress-related hormones like cortisol and adrenocorticotropic hormone (ACTH) underlie circadian rhythms (e.g., Dickmeis, 2009; Lightman and Conway-Campbell, 2010; Conway-Campbell et al., 2012), which could influence the potential time-of-day-dependence of tinnitus (loudness and distress). Thus, we also explored whether the stress-level as assessed with TYT depends on time-of-day and whether the 24-h fluctuations of tinnitus loudness and tinnitus distress change when taking the stress-level into account.

## Materials and methods

The material and the methods were approved by the Ethics Committee of the University Clinic of Regensburg and were carried out in accordance with the approved guidelines. Information that the TYT data will be used for scientific analyses is included in the mobile applications of “TrackYourTinnitus” as well as on the “TrackYourTinnitus” website and, therefore, the TYT users were informed that the data will be used for scientific purposes. Written consent, however, was not possible to obtain given the nature of the study. The study participants were anonymized.

### “TrackYourTinnitus” platform

The TYT platform (www.trackyourtinnitus.org, Pryss et al., 2015a,b) consists of a website for registration, two mobile applications (for iOS and Android), and a MySQL database as a central repository for the data collected. Users can either use TYT whenever they want or they can set a user-defined schedule to receive random notifications. For the study at hand, only these notification-triggered assessments were investigated. At each of these notifications, the users are asked to rate their tinnitus and other tinnitus-related variables (e.g., subjective stress-level). Although the attention might be directed toward the tinnitus by such notifications, Henry et al. (2012) and Schlee et al. (2016) found that repeatedly rating tinnitus and associated variables in daily life does not have detrimental effects. The present study investigated the following variables the users were asked to rate at each notification: “Current tinnitus loudness” (subjective rating of current tinnitus loudness on a visual analog scale [VAS], including a zero value for moments without loudness: min: 0; max: 1), “current tinnitus distress” (subjective rating of current tinnitus distress on a VAS including a zero value for moments without distress: min: 0; max: 1), and “current stress-level” (subjective rating of current stress-level on a VAS: min: 0; max: 1). Moreover, the timestamps of the assessments were used to explore the time-of-day-dependence of tinnitus. In TYT, the timestamps represent the local time of the time zone a given user is in when providing the assessments.

The data set used for the current study was exported in June 2016. After excluding the self-initiated assessments and the assessments given within the 15 min after the last assessment (for the inter-assessment interval of 15 min see also Pryss et al., 2015b), we had access to 25,863 notification-triggered assessments. For the present study, we only included the 25,092 assessments without missing values in any of the three target variables. Furthermore, as we were interested in within-day variations, we only considered data from days with at least three completed assessments, resulting in a total number of 17,209 assessments.

### Sample

The final sample consisted of 350 participants. Two-hundred and fifty three participants (72.2%) were male, 94 (26.9%) were female, and 3 did not indicate their gender. On average, participants were 45.4 (*SD* = 12.1) years old (17 participants did not report their age). The median number of years since onset of tinnitus was 5.4, ranging from 0 to 61.8 years. According to participants, onset of tinnitus was related to loud blast of sound (*n* = 48), whiplash (*n* = 9), change in hearing (*n* = 38), stress (*n* = 99), head trauma (*n* = 12), and other causes (*n* = 141) (3 participants did not report events related to onset of tinnitus). The median number of days per participant (with at least three assessments) was 11, ranging from 1 to 415 days. This corresponds to a total number of 3,570 days. The median number of assessments per day was 4, ranging from 3 (the minimum requirement to be included in this study) to 18 assessments.

### Statistical analyses

To test our hypotheses, we used multilevel modeling (MLM; Raudenbush and Bryk, 2002; Singer and Willett, 2003). MLM is ideally suited to address the nested structure of our data, with assessments (Level 1) nested in days (Level 2), and days nested in participants (Level 3). First, we estimated two MLMs predicting tinnitus loudness and tinnitus distress from time-of-day, respectively. Time-of-day was dummy-coded using five binary variables indicating whether the assessment was in the early morning (T1, from 4 a.m. to 8 a.m.), in the late morning (T2, from 8 a.m. to 12 p.m.), in the afternoon (T3, from 12 p.m. to 4 p.m.), in the early evening (T4, from 4 p.m. to 8 p.m.), or in the late evening (T5, from 8 p.m. to 12 a.m.). Assessments during the night (from 12 a.m. to 4 a.m.) were defined as the reference group. The full three-level MLM with random intercepts at Level 2 and 3 and random slopes at Level 3 (Model I) is summarized below:

**Table:** 

Level 1:	*y*_*ijk*_ = π_0*jk*_ + π_1*jk*_(*T*1) + π_2*jk*_(*T*2) + π_3*jk*_(*T*3)
	+ π_4*jk*_ (*T*4) + π_5*jk*_ (*T*5) + *e*_*ijk*_
Level 2:	π_0*jk*_ = β_00*k*_ + *r*_0*jk*_
	π_1*jk*_ = β_10*k*_
	π_2*jk*_ = β_20*k*_
	π_3*jk*_ = β_30*k*_
	π_4*jk*_ = β_40*k*_
	π_5*jk*_ = β_50*k*_
Level 3:	β_00*k*_ = γ_000_ + *u*_00*k*_
	β_10*k*_ = γ_100_ + *u*_10*k*_
	β_20*k*_ = γ_200_ + *u*_20*k*_
	β_30*k*_ = γ_300_ + *u*_30*k*_
	β_40*k*_ = γ_400_ + *u*_40*k*_
	β_50*k*_ = γ_500_ + *u*_50*k*_

The model decomposes the amount of tinnitus loudness/distress (*y*) of participant *k* on day *j* at assessment *i* into a series of fixed and random effects. The fixed effect γ_000_ represents the expected (population) tinnitus loudness/distress during the night before an average day of an average participant. The fixed effects γ_100_, γ_200_, γ_300_, γ_400_, and γ_500_ represent the expected change in tinnitus loudness/distress from night to early and late morning, afternoon and early and late evening, respectively. The random effects at Level 3, *u*_00*k*_, *u*_10*k*_, *u*_20*k*_, *u*_30*k*_, *u*_40*k*_, and *u*_50*k*_, indicate that the level of tinnitus loudness/distress at night as well as its later change during the day may differ between participants. The random intercept at Level 2, *r*_0*jk*_, indicates that the baseline level of tinnitus loudness/distress may differ between days within participants. We assumed random effects to be multivariate normally distributed within levels, and residuals to be independent and identically distributed across levels.

Second, we estimated the same MLM for stress-level as the dependent variable (y). Moreover, we estimated two further MLMs predicting tinnitus loudness and tinnitus distress from time-of-day, this time including stress-level as an additional predictor. As stress-level varied across all three levels, we decomposed its variance into three separate mean-centered variables capturing variation of stress within days (S1), variation of stress within participants across days (S2), and variation of stress across participants (S3) prior to estimating the MLMs. The full three-level MLM with random intercepts at Level 2 and 3 and random slopes at Level 3 (Model II) is summarized below:

**Table:** 

Level 1:	*y*_*ijk*_ = π_0*jk*_ + π_1*jk*_ (*T*1) + π_2*jk*_ (*T*2) + π_3*jk*_ (*T*3)
	+ π_4*jk*_ (*T*4) + π_5*jk*_ (*T*5) + π_6*jk*_ (*S*1) + *e*_*ijk*_
Level 2:	π_0*jk*_ = β_00*k*_ + β_01*k*_ (*S*2) + *r*_0*jk*_
	π_1*jk*_ = β_10*k*_
	π_2*jk*_ = β_20*k*_
	π_3*jk*_ = β_30*k*_
	π_4*jk*_ = β_40*k*_
	π_5*jk*_ = β_50*k*_
	π_6*jk*_ = β_60*k*_
Level 3:	β_00*k*_ = γ_000_ + γ_001_ (*S*3) + *u*_00*k*_
	β_01*k*_ = γ_010_ + *u*_01*k*_
	β_10*k*_ = γ_100_ + *u*_10*k*_
	β_20*k*_ = γ_200_ + *u*_20*k*_
	β_30*k*_ = γ_300_ + *u*_30*k*_
	β_40*k*_ = γ_400_ + *u*_40*k*_
	β_50*k*_ = γ_500_ + *u*_50*k*_
	β_60*k*_ = γ_600_ + *u*_60*k*_

The newly defined fixed effects, γ_001_, γ_010_, and γ_600_, represent the expected between-participant, between-day, and within-day effect of stress on tinnitus loudness/distress after controlling for time-of-day. The remaining fixed effects, γ_000_, γ_100_, γ_200_, γ_300_, γ_400_, and γ_500_, represent the expected level of tinnitus loudness/distress at night as well as its later change during the day after controlling for the influence of stress. The newly defined random effects, *u*_01*k*_ and *u*_60*k*_, indicate that the between- and within-day effects of stress may differ between participants. Due to model identification issues, we restricted the covariances between random effects of stress and the remaining random effects in the model to be zero.

All models were estimated using full maximum likelihood estimation. Analyses were conducted with the package “lme4” (Bates et al., 2015) of the statistical platform R (R Core Team, 2015). We used Satterthwaite's approximations to derive p-values for fixed effects. Pairwise comparisons between the six distinct timeframes were explored using the Tukey Honest Significant Difference method as implemented in the package “multcomp” (Hothorn et al., 2008). Finally, we quantified the effect size of time-of-day on tinnitus loudness/distress by means of a pseudo *R*^2^ statistic. This statistic can be computed by subtracting the residual variance *Var*(*e*_*ijk*_) of Model I from the residual variance of an intercept-only model without any predictors, divided by this latter residual variance. It represents the relative amount of variance in tinnitus loudness/distress within days that is explained by time-of-day (i.e., by the five variables T1–T5).

## Results

**Figure 1:**
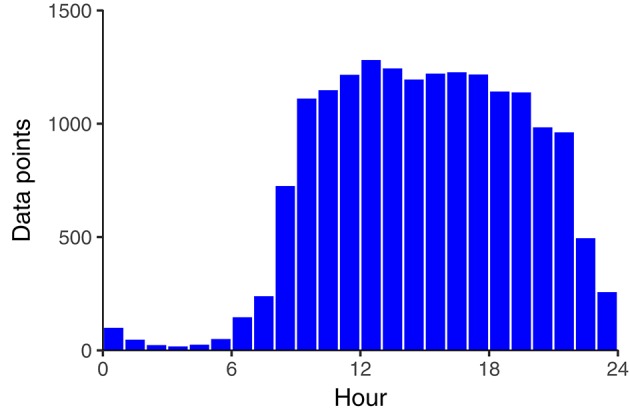
Number of data points per hour.

**Figure 2:**
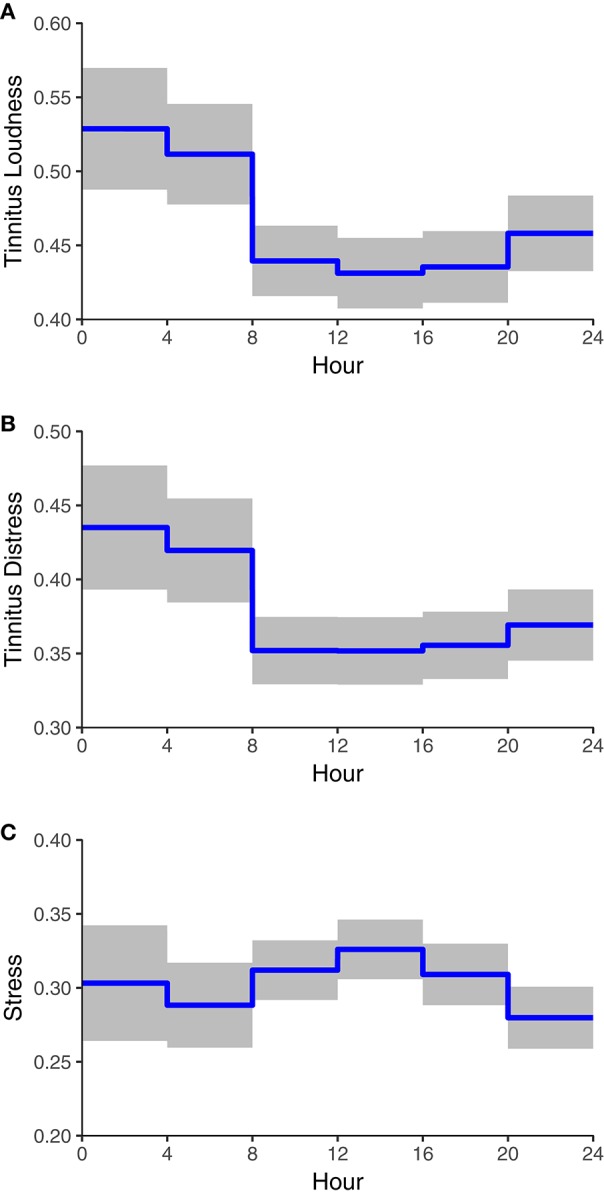
Expected tinnitus loudness **(A)**, tinnitus distress **(B)**, and level of stress **(C)** as a function of time-of-day. The blue lines represent expected values for the average participant on an average day, the gray bands represent 95% confidence intervals.

In total, 186 assessments (1.1%) were completed at night, 460 (2.7%) were completed in the early morning, 4,200 (24.4%) were completed in the late morning, 4,941 (28.7%) in the afternoon, 4,724 (27.5%) in the early evening, and 2,698 (15.7%) were completed in the late evening (see Figure 1). Table 1 summarizes the estimated fixed effects of all MLMs (standard deviations and correlations of random effects can be found in the Supplementary Material). Results suggest that tinnitus was louder and more distressing during the night and early morning hours than during all other timeframes of the day (see Models I in the first and third columns of Table 1). Tukey's post-hoc tests revealed that differences between late morning, afternoon, and early evening were non-significant (see Table 2). However, tinnitus was significantly louder in the late evening compared to the afternoon and early evening. This pattern of results is visualized in Figures 2A,B. The pseudo R^2^ statistics revealed that time-of-day explained 20.6% of the within-day variance of tinnitus loudness, and 13.0% of the within-day variance of tinnitus distress.

**Table 1:** Estimated fixed effects (and their standard errors) for five MLMs.

		**Tinnitus loudness**		**Tinnitus distress**		**Stress-level**
		**Model I**	**Model II**	**Model I**	**Model II**	**Model I**
Intercept (night)	γ_000_	0.529^***^ (0.021)	0.521^***^ (0.018)	0.435^***^ (0.021)	0.423^***^ (0.016)	0.303^***^ (0.020)
Early morning (vs. night) effect	γ_100_	−0.017 (0.021)	−0.015 (0.019)	−0.015 (0.022)	−0.014 (0.018)	−0.015 (0.020)
Late morning (vs. night) effect	γ_200_	−0.089^***^ (0.019)	−0.086^***^ (0.017)	−0.083^***^ (0.020)	−0.077^***^ (0.016)	0.009 (0.019)
Afternoon (vs. night) effect	γ_300_	−0.098^***^ (0.019)	−0.099^***^ (0.017)	−0.083^***^ (0.019)	−0.083^***^ (0.016)	0.023 (0.018)
Early evening (vs. night) effect	γ_400_	−0.093^***^ (0.019)	−0.092^***^ (0.017)	−0.080^***^ (0.020)	−0.074^***^ (0.015)	0.006 (0.018)
Late evening (vs. night) effect	γ_500_	−0.071^***^ (0.019)	−0.062^***^ (0.016)	−0.066^***^ (0.019)	−0.052^***^ (0.014)	−0.023 (0.018)
Within-day effect of stress	γ_600_		0.287^***^ (0.020)		0.370^***^ (0.022)	
Between-day effect of stress	γ_010_		0.470^***^ (0.037)		0.585^***^ (0.035)	
Between-person effect of stress	γ_001_		0.651^***^ (0.051)		0.810^***^ (0.041)	

*Total number of assessments was 17,209. p-values were based on Satterthwaite's approximations*.

*** *p < 0.001. night = 12 a.m.–4 a.m. early morning = 4 a.m.–8 a.m. late morning = 8 a.m.–12 p.m. afternoon = 12 p.m.–4 p.m. early evening = 4 p.m.–8 p.m. late evening = 8 p.m.–12 a.m*.

**Table 2:** Tukey's *post-hoc* tests for five MLMs.

	**Tinnitus loudness**		**Tinnitus distress**		**Stress-level**
	**Model I**	**Model II**	**Model I**	**Model II**	**Model I**
Early_morning–night	−0.017 (0.021)	−0.015 (0.019)	−0.015 (0.022)	−0.014 (0.018)	−0.015 (0.020)
Late_morning–night	−0.089^***^ (0.019)	−0.086^***^ (0.017)	−0.083^***^ (0.020)	−0.077^***^ (0.016)	0.009 (0.019)
Afternoon–night	−0.098^***^ (0.019)	−0.099^***^ (0.017)	−0.083^***^ (0.019)	−0.083^***^ (0.016)	0.023 (0.018)
Early_evening–night	−0.093^***^ (0.019)	−0.092^***^ (0.017)	−0.080^***^ (0.020)	−0.074^***^ (0.015)	0.006 (0.018)
Late_evening–night	−0.071^**^ (0.019)	−0.062^**^ (0.016)	−0.066^**^ (0.019)	−0.052^**^ (0.014)	−0.023 (0.018)
Late_morning–early_morning	−0.072^***^ (0.014)	−0.071^***^ (0.013)	−0.068^***^ (0.015)	−0.063^***^ (0.013)	0.023 (0.012)
Afternoon–early_morning	−0.080^***^ (0.016)	−0.084^***^ (0.015)	−0.068^***^ (0.016)	−0.069^***^ (0.014)	0.038^*^ (0.012)
Early_evening–early_morning	−0.076^***^ (0.016)	−0.077^***^ (0.015)	−0.064^***^ (0.016)	−0.060^***^ (0.014)	0.021 (0.013)
Late_evening–early_morning	−0.053^**^ (0.016)	−0.047^*^ (0.015)	−0.050^*^ (0.017)	−0.038^*^ (0.014)	−0.009 (0.012)
Afternoon–late_morning	−0.008 (0.005)	−0.013^#^ (0.005)	−0.000 (0.005)	−0.006 (0.004)	0.014^**^ (0.004)
Early_evening–late_morning	−0.004 (0.007)	−0.005 (0.006)	0.004 (0.006)	0.003 (0.004)	−0.003 (0.006)
Late_evening–late_morning	0.019 (0.009)	0.025^*^ (0.008)	0.017 (0.008)	0.025^**^ (0.007)	−0.032^***^ (0.006)
Early_evening–afternoon	0.004 (0.003)	0.007 (0.003)	0.004 (0.004)	0.009^*^ (0.003)	−0.017^***^ (0.004)
Late_evening–afternoon	0.027^**^ (0.007)	0.037^***^ (0.007)	0.017 (0.007)	0.031^***^ (0.006)	−0.046^***^ (0.005)
Late_evening–early_evening	0.023^**^ (0.006)	0.030^***^ (0.006)	0.014 (0.006)	0.022^***^ (0.005)	−0.029^***^ (0.004)

*Total number of assessments was 17,209. The table presents estimated differences (and their standard errors) for all comparisons between timeframes. p-values were adjusted using the Tukey Honest Significant Difference method*.

# *p < 0.10*.

* *p < 0.05*.

** *p < 0.01*.

*** *p < 0.001. night = 12 a.m.–4 a.m. early morning = 4 a.m.–8 a.m. late morning = 8 a.m.–12 p.m. afternoon = 12 p.m.–4 p.m. early evening = 4 p.m.–8 p.m. late evening = 8 p.m.–12 a.m*.

Next, we tested whether time-of-day influences the subjective stress-level (see Model I in the right hand column of Table 1 and Figure 2C). Tukey's post-hoc tests suggested that stress-level increased from morning to afternoon, decreased from afternoon to evening, and did not differ compared to the night (see Table 2). Time-of-day explained 7.8% of the within-day variance of stress.

Finally, the models predicting tinnitus loudness and tinnitus distress from time-of-day and stress-level revealed that the stress-level had incremental effects across all three levels (see Models II in Table 1): Tinnitus was louder and more distressing when the level of stress was higher at a specific time-of-day compared to other times-of-day, when it was higher during a whole day compared to other days, and when it was higher during the whole assessment period for a given participant (compared to other participants). Nevertheless, the effects of time-of-day on tinnitus loudness and tinnitus distress were still significant (i.e., after controlling for the effects of stress).

## Discussion

This study evaluated whether subjective tinnitus loudness and subjective tinnitus distress depend on time-of-day with EMA from the “TrackYourTinnitus” (TYT) mobile application (Pryss et al., 2015a,b; Schlee et al., 2016). Strengths of the present study are that a mobile application was used to obtain EMA of high ecological validity (Trull and Ebner-Priemer, 2013, 2014; Adams et al., 2017), a much higher sampling frequency was applied than in typical clinical studies, and the sample size was much larger than in previous EMA studies on the time-of-day-dependence of tinnitus (Flor et al., 2004; Henry et al., 2012; Wilson et al., 2015).

The main result was that tinnitus (loudness and distress) was rated as more severe during the night and the early morning (from 12 a.m. to 8 a.m.) than during the upcoming day. Interestingly, tinnitus loudness and tinnitus distress showed a very similar time pattern although the neurobiological correlates of loudness and distress differ to a certain degree (e.g., De Ridder et al., 2011; Leaver et al., 2012; Ueyama et al., 2013; Vanneste et al., 2014).

Contrary to the EMA study by Henry et al. (2012), the present investigation found a time-of-day-dependence of tinnitus. The most obvious reason for this discrepancy is that Henry et al. (2012) assessed tinnitus only from 8 a.m. to 8 p.m. and could, therefore, not evaluate tinnitus during the time interval that was related to most severe tinnitus in our study (12 a.m.–8 a.m.). During a time interval (8 a.m.–12 a.m.) that included the 12-h interval analyzed by Henry et al. (2012) tinnitus did not vary much in our study either (see Figures 2A,B). The result that tinnitus was more severe in the early morning hours is in line with a previous diary study (Flor et al., 2004) and fits to the clinical impression of a “morning roar” as it is often anecdotally reported by tinnitus patients. Yet, our result that tinnitus was more severe during the night was not found by Flor et al. (2004). More severe tinnitus (loudness and distress) during the night could at least partially result from the possibility that several assessments at night were given by participants while having sleep disturbances. Sleep disturbances are common among tinnitus patients and their severity correlates positively with measures of tinnitus severity (e.g., Crönlein et al., 2007; Schecklmann et al., 2015; Crönlein et al., 2016). Although we aimed to control for this potential confounder by excluding data from spontaneous ratings and restricting the data analysis to the ratings provided at notifications, the fact that sleep disturbances were not assessed on a daily basis in the present study and, hence, were not available for analysis is a limitation of our study. Future research could explore whether the effect of time-of-day on tinnitus is different between days with and without sleep disturbances. Besides sleep, several other variables are time-of-day dependent and should be considered as potential confounding variables: For example, the environmental sound level depends on time-of-day and tinnitus might be more severe during the night and the early morning, because the environmental sound level in these time intervals cannot mask the tinnitus. Furthermore, a trigger of tinnitus might be (even slight) tension of specific muscles of the craniocervical connection (Bechter et al., 2016), whereby the tension depends mainly from posture, which is influenced by different aspects during the night (e.g., sleeping posture, bed pillow) and during the day. Moreover, the release of stress-related hormones is controlled by a circadian clock (Dickmeis, 2009; Lightman and Conway-Campbell, 2010; Conway-Campbell et al., 2012) and future studies could explore the relationship between circadian tinnitus variability and such stress-related hormones. In the present study, we could only analyze subjective stress-levels with the result that the 24-h course of the subjective stress-level was not parallel to the course of tinnitus (loudness and distress): While tinnitus (loudness and distress) showed their maximum between 12 a.m. and 8 a.m., the maximum of the subjective stress-level was found between 12 p.m. and 4 p.m. The discrepant time curves of tinnitus and subjective stress-level raise further questions, which need to be addressed in future longitudinal studies regarding the tinnitus-stress link. For example, it appears possible that tinnitus and subjective stress interact more in a time-lagged than in an instant manner. Thus, it should be clarified whether increases of subjective stress lead to more severe tinnitus and whether increases of tinnitus severity also lead to more subsequent subjective stress (which again might affect tinnitus severity resulting in a vicious circle). Feedback loops have been described already in research on the pituitary-adrenal system: “positive, delayed, feedforward connection between the pituitary and the adrenals” and “negative feedback of glucocorticoids on ACTH release” (Lightman and Conway-Campbell, 2010, p. 712). It should also be noted in this context that the peaks of our self-reported stress-levels (12 p.m.–4 p.m.) did not correspond to the peaks of cortisol and ACTH releases (4 a.m.–8 a.m.) as illustrated by Lightman and Conway-Campbell (2010). Therefore, the longitudinal associations between tinnitus and self-reported stress might be different from the ones between tinnitus and stress-hormones.

Regarding the observed circadian variability of tinnitus loudness, it is tempting to speculate relations to circadian variations in neuronal sensitivity of peripheral and central auditory structures, which have been identified in recent studies (Park et al., 2016; Basinou et al., 2017). However, tinnitus loudness does not directly reflect neuronal activity in auditory pathways, but also depends on other factors such as attention and emotions. For example, a previous study found that tinnitus becomes louder over time when participants experience more qualitatively different feelings (Probst et al., 2016b). Therefore, more research is needed to replicate the observed circadian rhythm of tinnitus in large samples, to identify the specific contribution of different factors (e.g., neuronal activity in auditory pathways, attention, and emotion), and to explore whether different persons show a different time-of-day dependence of tinnitus. Moderating variables might, for example, be gender, age (e.g., the time-of-day dependence of tinnitus might be different between men and women or within women before/after the menopause), and the etiology of tinnitus (e.g., the time of time-of-day dependence might be different between noise trauma as etiology and stress as etiology). Future translational and clinical studies with samples large enough to analyze such subgroups are required to explore potential moderators of the time-of-day dependence of tinnitus. These further studies could also measure tinnitus by different assessment methods, because the study at hand only analyzed self-reported tinnitus. For example, by the “gap-in-noise” paradigm (Lowe and Walton, 2015) or psychophysiological measurements such as tinnitus matching. Yet, the reliability and validity of tinnitus matching is an ongoing matter of debate (Hoare et al., 2014; De Ridder et al., 2015). For mobile applications such as TYT, portable methods to assess tinnitus more objectively (see for example, Hébert and Fournier, 2017) that can be integrated in the mobile application are needed. It is necessary to evaluate whether a similar time-of-day dependence of tinnitus can be shown for self-reported tinnitus as well as for more objectively measured tinnitus. Future studies could also compare different statistical approaches and identify the approach most suited to investigate time-of-day dependencies. For example, harmonic regression was performed in an animal study on circadian rhythm (Atger et al., 2015), and an R package for nonparametric circular methods (NPCirc) is available to analyze circular data (Oliveira et al., 2014). These approaches were not performed in the present study due to the nested structure of the data (assessments within days and days within participants).

In summary, the results of our study have implications for both tinnitus research and tinnitus management. Taking time-of-day into account in the study design might be necessary in clinical studies. Yet, the results of our study rely solely on TYT users (who are not representative for clinical tinnitus patients, see Probst et al., 2017) and on one-item questions on tinnitus distress and tinnitus loudness making it difficult to conclude whether the observed differences between night/early morning and the upcoming day are clinically relevant. Future studies using assessment instruments appropriate to distinguish between clinically relevant and clinically irrelevant change (for example the Tinnitus Handicap Inventory (THI), see Zeman et al., 2011) with clinical tinnitus samples are necessary to address this point. Chronobiological aspects should not only be considered in tinnitus research but also in tinnitus treatment. Tailoring the timing of therapeutic interventions to the circadian rhythm of individual tinnitus patients (chronotherapy) might be promising. Further translational and clinical studies are necessary to evaluate the potential of chronotherapy for tinnitus. However, this is a challenging task since “research in the topic is underfunded when compared with other diseases for which the prevalence and cost to society is relatively similar” (Cederroth et al., 2013, p. 972). Nevertheless, the current study might motivate individuals with tinnitus to observe their tinnitus fluctuations and to identify time-intervals during which the access to and the use of tinnitus coping strategies are most crucial.

## Author contributions

TP substantially contributed to the design of the study and data preparation, drafted and revised the manuscript. RP substantially contributed to the design of the study, data preparation, conception, implementation and maintenance of the “TrackYourTinnitus” application, and revised the manuscript. BL substantially contributed to the design of the study and revised the manuscript. JR substantially contributed to the design of the study and revised the manuscript. JS substantially contributed to the conception, implementation and maintenance of the “TrackYourTinnitus” application, and revised the manuscript. MR substantially contributed to the conception, implementation and maintenance of the “TrackYourTinnitus” application, and revised the manuscript. MS substantially contributed to the design of the study and revised the manuscript. WS substantially contributed to the design of the study, data preparation, conception and implementation of the “TrackYourTinnitus” application, drafted and revised the manuscript. JZ substantially contributed to the design of the study and performed the statistical analysis, drafted and revised the manuscript.

### Conflict of interest statement

The other authors declare that the research was conducted in the absence of any commercial or financial relationships that could be construed as a potential conflict of interest. The handling Editor declared a co-authorship with the authors TP, RP, BL, MR, MS and WS, and the handling Editor states that the process met the standards of a fair and objective review.
