# Supplementary material for: Does Tinnitus Depend on Time-of-Day? An Ecological Momentary Assessment Study with the “TrackYourTinnitus” Application
Source: Front Aging Neurosci. 2017 Aug 2;9:253. doi: 10.3389/fnagi.2017.00253 (PMC5539131; doi:10.3389/fnagi.2017.00253)
Supplement: Supplementary file 1 [file Table1.docx]

Supplementary material

Table 1

*Standard deviations and correlations of random effects of Model I predicting tinnitus loudness*

|  |  | *SD* | $r_{.u_{00k}}$ | $r_{.u_{10k}}$ | $r_{.u_{20k}}$ | $r_{.u_{30k}}$ | $r_{.u_{40k}}$ |
| --- | --- | --- | --- | --- | --- | --- | --- |
| level 2 intercept | $r_{0jk}$ | 0.11 |  |  |  |  |  |
| level 3 intercept | $u_{00k}$ | 0.23 |  |  |  |  |  |
| level 3 slope early morning | $u_{10k}$ | 0.15 | -.43 |  |  |  |  |
| level 3 slope late morning | $u_{20k}$ | 0.16 | -.52 | .65 |  |  |  |
| level 3 slope afternoon | $u_{30k}$ | 0.15 | -.48 | .38 | .93 |  |  |
| level 3 slope early evening | $u_{40k}$ | 0.15 | -.46 | .32 | .86 | .98 |  |
| level 3 slope late evening | $u_{50k}$ | 0.14 | -.42 | .33 | .72 | .82 | .90 |
| level 1 residual | $e_{ijk}$ | 0.11 |  |  |  |  |  |

*Note*. Total number of assessments was 17,209.

Table 2

*Standard deviations and correlations of random effects of Model I predicting tinnitus distress*

|  |  | *SD* | $r_{.u_{00k}}$ | $r_{.u_{10k}}$ | $r_{.u_{20k}}$ | $r_{.u_{30k}}$ | $r_{.u_{40k}}$ |
| --- | --- | --- | --- | --- | --- | --- | --- |
| level 2 intercept | $r_{0jk}$ | 0.10 |  |  |  |  |  |
| level 3 intercept | $u_{00k}$ | 0.24 |  |  |  |  |  |
| level 3 slope early morning | $u_{10k}$ | 0.17 | -.45 |  |  |  |  |
| level 3 slope late morning | $u_{20k}$ | 0.19 | -.60 | .72 |  |  |  |
| level 3 slope afternoon | $u_{30k}$ | 0.17 | -.57 | .59 | .95 |  |  |
| level 3 slope early evening | $u_{40k}$ | 0.17 | -.57 | .59 | .94 | .98 |  |
| level 3 slope late evening | $u_{50k}$ | 0.15 | -.53 | .47 | .87 | .86 | .92 |
| level 1 residual | $e_{ijk}$ | 0.11 |  |  |  |  |  |

*Note*. Total number of assessments was 17,209.

Table 3

*Standard deviations and correlations of random effects of Model I predicting level of stress*

|  |  | *SD* | $r_{.u_{00k}}$ | $r_{.u_{10k}}$ | $r_{.u_{20k}}$ | $r_{.u_{30k}}$ | $r_{.u_{40k}}$ |
| --- | --- | --- | --- | --- | --- | --- | --- |
| level 2 intercept | $r_{0jk}$ | 0.10 |  |  |  |  |  |
| level 3 intercept | $u_{00k}$ | 0.22 |  |  |  |  |  |
| level 3 slope early morning | $u_{10k}$ | 0.15 | -.60 |  |  |  |  |
| level 3 slope late morning | $u_{20k}$ | 0.17 | -.65 | .84 |  |  |  |
| level 3 slope afternoon | $u_{30k}$ | 0.14 | -.62 | .78 | .99 |  |  |
| level 3 slope early evening | $u_{40k}$ | 0.13 | -.59 | .66 | .93 | .95 |  |
| level 3 slope late evening | $u_{50k}$ | 0.13 | -.61 | .75 | .94 | .94 | .98 |
| level 1 residual | $e_{ijk}$ | 0.12 |  |  |  |  |  |

*Note*. Total number of assessments was 17,209.

Table 4

*Standard deviations and correlations of random effects of Model II predicting tinnitus loudness when statistically controlling for level of stress*

|  |  | *SD* | $r_{.u_{00k}}$ | $r_{.u_{10k}}$ | $r_{.u_{20k}}$ | $r_{.u_{30k}}$ | $r_{.u_{40k}}$ |
| --- | --- | --- | --- | --- | --- | --- | --- |
| level 2 intercept | $r_{0jk}$ | 0.09 |  |  |  |  |  |
| level 3 intercept | $u_{00k}$ | 0.20 |  |  |  |  |  |
| level 3 slope early morning | $u_{10k}$ | 0.11 | -0.26 |  |  |  |  |
| level 3 slope late morning | $u_{20k}$ | 0.13 | -0.54 | 0.53 |  |  |  |
| level 3 slope afternoon | $u_{30k}$ | 0.13 | -0.54 | 0.25 | 0.91 |  |  |
| level 3 slope early evening | $u_{40k}$ | 0.13 | -0.52 | 0.19 | 0.85 | 0.98 |  |
| level 3 slope late evening | $u_{50k}$ | 0.12 | -0.46 | 0.18 | 0.65 | 0.79 | 0.88 |
| level 3 slope within-day stress | $u_{60k}$ | 0.24 |  |  |  |  |  |
| level 3 slope between-day stress | $u_{01k}$ | 0.34 |  |  |  |  |  |
| level 1 residual | $e_{ijk}$ | 0.11 |  |  |  |  |  |

*Note*. Total number of assessments was 17,209.

Table 5

*Standard deviations and correlations of random effects of Model II predicting tinnitus distress when statistically controlling for level of stress*

|  |  | *SD* | $r_{.u_{00k}}$ | $r_{.u_{10k}}$ | $r_{.u_{20k}}$ | $r_{.u_{30k}}$ | $r_{.u_{40k}}$ |
| --- | --- | --- | --- | --- | --- | --- | --- |
| level 2 intercept | $r_{0jk}$ | 0.08 |  |  |  |  |  |
| level 3 intercept | $u_{00k}$ | 0.17 |  |  |  |  |  |
| level 3 slope early morning | $u_{10k}$ | 0.11 | -0.26 |  |  |  |  |
| level 3 slope late morning | $u_{20k}$ | 0.14 | -0.66 | 0.61 |  |  |  |
| level 3 slope afternoon | $u_{30k}$ | 0.15 | -0.69 | 0.44 | 0.96 |  |  |
| level 3 slope early evening | $u_{40k}$ | 0.13 | -0.67 | 0.46 | 0.95 | 0.99 |  |
| level 3 slope late evening | $u_{50k}$ | 0.09 | -0.63 | 0.30 | 0.86 | 0.91 | 0.94 |
| level 3 slope within-day stress | $u_{60k}$ | 0.29 |  |  |  |  |  |
| level 3 slope between-day stress | $u_{01k}$ | 0.33 |  |  |  |  |  |
| level 1 residual | $e_{ijk}$ | 0.10 |  |  |  |  |  |

*Note*. Total number of assessments was 17,209.
